# Stimulation of Epicardial Sympathetic Nerves at Different Sites Induces Cardiac Electrical Instability to Various Degrees

**DOI:** 10.1038/s41598-018-19304-2

**Published:** 2018-01-17

**Authors:** Liang Wang, Lin Sun, Kun Wang, Yingying Jin, Qing Liu, Zhongnan Xia, Xudong Liu, Jiakun Zhang, Jingjie Li

**Affiliations:** 1 The First Affiliated Hospital of Harbin Medical University, Harbin, China; 2 Henan Provincial Chest Hospital, Henan, China

## Abstract

The cardiac sympathetic nerves distribute across cardiac tissues with uneven density. Yet, to what extent this anatomical heterogeneity affects electrical activity of the left ventricle is largely unknown. Dogs were randomized into non-stimulation control (NC), posterior basal-stimulation (PB), anterior superior-stimulation (AS), apical part-stimulation (AP) group. The epicardial sympathetic nerves at different sites along their distribution were with electrical stimulation (ES) for 4 hours except in the NC group. The myocardial effective refractory period (ERP), ventricular fibrillation threshold (VFT) and density of sympathetic nerves were recorded. Compared with ES at other places, the stimulation at PB site significantly shortened ERP (left ventricular anterior and posterior walls; PB group, 118 ± 4 ms, 106 ± 2 ms; Versus NC group, 155 ± 3.5 ms, 160 ± 3 ms; *p* < 0.01) and VFT (PB group, 11.5 ± 1.5 V; Versus NC group, 20.5 ± 0.9 V; *p* < 0.01), and induced remarkable regeneration of the cardiac sympathetic nerves, hence influencing electrical activity of the left ventricle to the most extent. Our study demonstrates that the degree of induced ventricular electrical instability is correlated tightly with the density of sympathetic nerves around ES site, and PB site is a potential target for modulating ventricular electrical activity to the maximal extent.

## Introduction

The cardiac sympathetic nerves arise from stellate ganglia, and innervate in cardiac tissues following coronary veins and arteries during heart development^1^. The uneven distribution of coronary vessels across cardiac tissue and the anatomical congruence between sympathetic nerve fibers and coronary vessels result in the heterogeneous innervation of sympathetic nerves^2^. The cardiac sympathetic nerves are required for maintaining normal electrical activity, but to what extend their anatomical heterogeneity affects cardiac electrical activity is largely unknown. Addressing this question would provide clinical implications in managing heart diseases that were resulted from the over-activation of cardiac sympathetic nerves.

The hyperactivity of cardiac sympathetic nerves has been linked to ventricular arrhythmias (VAs) and sudden cardiac death (SCD) in MI patients under physiological conditions. β-adrenergic receptor blockade inhibits the activity of cardiac sympathetic nerve and decreases the incidence of SCD^3,4^. Our previous study demonstrated that local ablation of the coronary sinus (CS) and great cardiac vein (GCV) peripheral nerve reduces VAs in a canine AMI model, suggesting that modulating the activity of cardiac sympathetic nerves may shed light to manage VAs and to prevent sudden cardiac death^5^. Due to heterogeneous distribution of nerve fibers in cardiac tissue, targeting the cardiac sympathetic nerves at an appropriate location would be critical for success of this regimen. To determine a site at which modulating the cardiac sympathetic nerves affects the left ventricular electrical activity to the maximal extent, in the present study, we stimulated the epicardial sympathetic nerves at the different sites based on their anatomical distribution. Our results indicate that stimulating the epicardial sympathetic nerves at the posterior basal area induces the most dramatic alterations in cardiac electrical stability.

## Materials and Methods

### Animal Preparation

Healthy male dogs were obtained from the experimental animal center at the First Affiliated Hospital of Harbin Medical University. All procedures related to animal experiments were conducted as per the institutional animal care guidelines and ethically approved by the Administration Committee for Experimental Animals at the First Affiliated Hospital of Harbin Medical University.

Sixteen dogs, weighing between 15 and 25 kg, were anesthetized with sodium pentobarbital (25 mg/kg body weight-induction; 1.0 mg/kg/h with intermittent boluses). During the experiment, the animals were intubated and mechanically ventilated (Electrical Animal Ventilator, Medical Equipment Factory, Shanghai, China) to maintain the arterial pCO_2_ between 35 and 40 mmHg. Fluid resuscitation was also established with 0.9 N NaCl at 10 ml/kg/h. The Systolic Blood Pressure (SBP) was monitored with a computer-based Lab System (GY-6328, Huanan Inc., China). The core body temperature was maintained at 36.5 ± 1.5 °C with heating pads. All animals received continuous ECG recording, and underwent thoracotomy and pericardiotomy.

### Determination of ES sites

During heart development, the coronary veins guide the innervation of sympathetic nerves. The physical proximity and the similar branching pattern of cardiac vessels and sympathetic nerve fibers cause the distributional heterogeneity of sympathetic nerves in cardiac tissue^2^. Based on this anatomical characteristic of the cardiac sympathetic nerves, we located the nerve fibers by following the distribution of blood vessels, which are easily recognized by the naked eye. We then validated sympathetic nerves with ES and subsequent alterations of SBP. The SBP increased by 20 mmHg or more is an indicator of the activation of sympathetic nerves^6^. We chose three sites, posterior basal, anterior superior and apical site, on the epicardium of the left ventricle for ES. These three locations are supposed to be reflective of the density of sympathetic nerves in ventricular tissues from high to low, respectively.

### Sympathetic Nerve Stimulation (SNS) and Cardiac Electrophysiology

**Figure 1:**
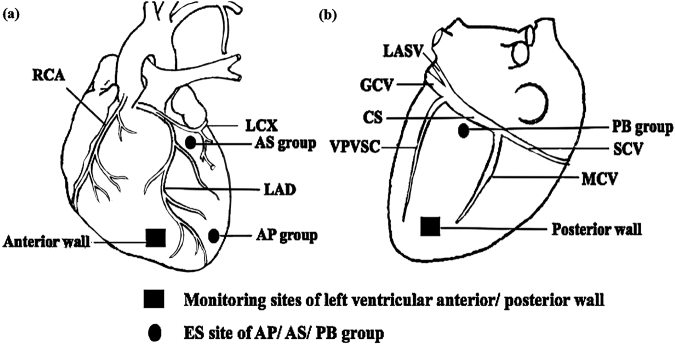
A diagram of the left ventricular anterior (**a**) and posterior (**b**) walls of a dog’s heart to show ES and electrophysiology-monitoring sites. LAD, left anterior descending coronary artery; LCX, left circumflex coronary artery; CS, coronary sinus; GCV, great cardiac vein; MCV, middle cardiac vein; SCV, small cardiac vein; LASV, left atrium slanting veins; VPVSC, vena-posterior-ventriculi-sinistri-cordis.

Based on the epicardial sites of ES, 16 animals were randomized into four groups, non-stimulation control (NC), posterior basal site-stimulation (PB), anterior superior site-stimulation (AS), and apical part-stimulation (AP) groups (Fig. 1).

Electrophysiological bipolar catheters (Irvine Biomedical Inc. USA) contained the distal and proximal electrodes with 2 mm pole spacing. The tips of electrodes were fixed on epicardium at the AV node, ES and monitoring sites. Four bipolar catheters per animal were used. One catheter was for pacing at the AV node, one for ES and others for monitoring electrical activity. To avoid inadvertent ES of the ventricles, ES within ERP (train duration, 50 ms; frequency, 200 Hz; 37.5 V; 2 ms pulse duration) was coupled with the pacing stimulus (140 bpm with a delay of 20 ms). All of the animals in the PB, AS and AP groups, not in NC group, underwent ES for 4 hours. The efferent SNS response during ES was determined by a 20 mmHg increase in SBP^6^.

Transition of epicardial ERP prior to and post ES at the monitoring sites (Fig. 1) was recorded. Briefly, an 8-beat drive train (S1, 300-ms cycle length; 2 ms, pulse duration) was employed, and then followed with an extra stimulation (S2, 2 ms, pulse duration). This procedure was repeated with S1–S2 intervals progressively shorten by a step length of 5 ms from 250 ms to ventricular ERP. Ventricular ERP was defined as the longest S1–S2 interval that failed to capture the ventricle.

VFT, a minimum voltage to induce sustained VF^7^, was recorded in all animals. To eliminate variations in the vulnerability of the fibrillation associated with the slowing or accelerating heart rate, all VFT measurements were performed at the same heart rate. At the end of a 20-beat drive train with 300-ms of pacing cycle length, 100-ms S1–S1 stimulation was applied to the right ventricular apex with an intensity increased by 2V each time until VF was induced. Stimulation lasted for 10 seconds and was followed by a 30-second rest period before the next round of stimulation. Once a sustained VF was induced, a cardiac electric defibrillator was used to shock the heart back to normal rhythm. After a 5-minute break, the stimulation protocol was repeated to measure the second VFT. The measurements of both times were averaged as mean VFT.

β-receptor blocker (Esmolol, 1 mg/kg) was given to all animals after electrophysiological measurements, and then ERP and VFT were measured again following the same procedures as described above.

### Histology and Immunohistochemical staining

At the end of the experiment, the hearts were collected from all dogs. Tissues around the ES sites were fixed for at least 24 h in 10% formalin, and embedded in paraffin. Then, 4 μm-thick sections were cut, mounted on charged slides and stained with a standard protocol. Briefly, paraffin sections were deparaffinized and rehydrated. Antigen was retrieved with Target Unmasking Fluid (Advanced Technology & Industrial Co., Hong Kong) according to the manufacturer’s protocol. Sections were incubated overnight at 4 °C with tyrosine hydroxylase (TH) and choline acetyl-transferase (ChAT) antibody (1:1000, Abcam Ltd., Hong Kong; 1:800, Bioss Ltd., Beijing), washed and then incubated with secondary IgG-HRP (Zsbio, China) for 15 min at room temperature. The immunoreactive products were visualized with Liquid DAB Substrate-Chromogen System. TH is a marker of sympathetic nerves^8^. ChAT is the synthetic enzyme for acetylcholine, the main parasympathetic neurotransmitter^9^. We determined nerve density by a computer assisted image analysis system (Motic Images Advanced 3.2 software). The nerve density was calculated as the nerve area divided by the total area examined (μm^2^/mm^2^).

### Statistical analysis

The SPSS software (version 17.0, SPSS, USA) was used in statistical analysis. Comparisons among continuous data were performed with a one-way analysis of variance (ANOVA); whereas categorical data were analyzed with Chi-square test. A p-value less than 0.05 was considered statistically significant.

## Results

### ES and β-receptor blocker change SBP

**Figure 2:**
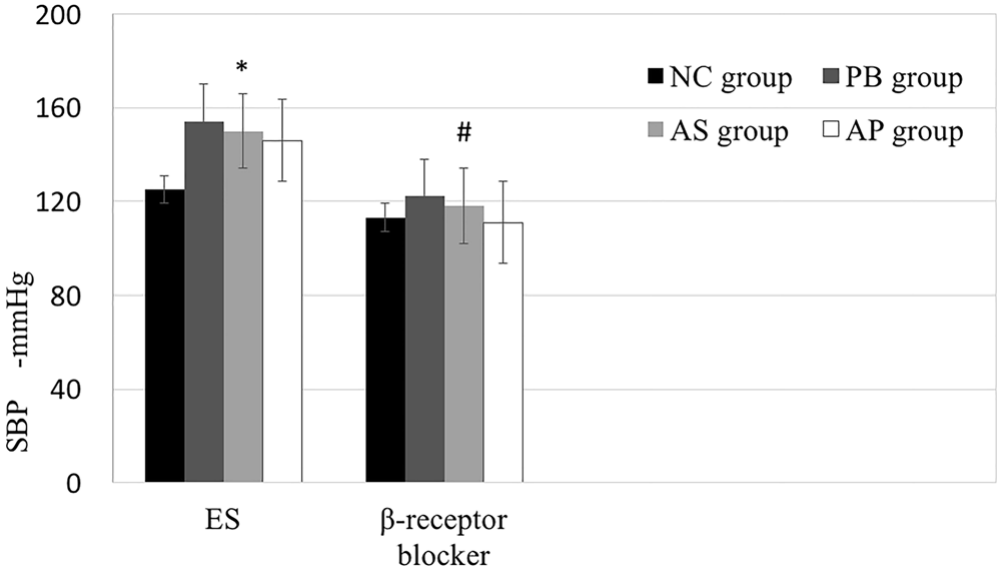
The Systolic Blood Pressure (SBP) was monitored after ES and β1-receptor blockade in PB, AS and AP groups. ^*^p < 0.05, compared to NC group.

ES at the different sites increased SBP significantly. Compared to NC group, SBP in PB, AS and AP groups increased by at least20 mmHg (PB group, 154 ± 7 mmHg, *p* = 0.0005; AS group, 150 ± 5 mmHg, *p* = 0.0006; AP group, 146 ± 6 mmHg, *p* = 0.002; Compared to NC group, 125 ± 5 mmHg; Fig. 2). β1-receptor blockade with Esmolol completely abolished the elevated SBP caused by ES (PB group, 122 ± 6 mmHg, *p* = 0.12; AS group, 118 ± 10 mmHg, *p* = 0.43; AP group, 111 ± 6 mmHg, *p* = 0.74; Compared to NC group, 113 ± 8 mmHg; Fig. 2). These results indicate that ES in the present study activates the cardiac sympathetic nerves. Although SBP increased by at least 20 mmHg after ES, SBP in the PB and AS groups increased much faster than that in the AP group.

### Assessment of cardiac electrophysiology

**Figure 3:**
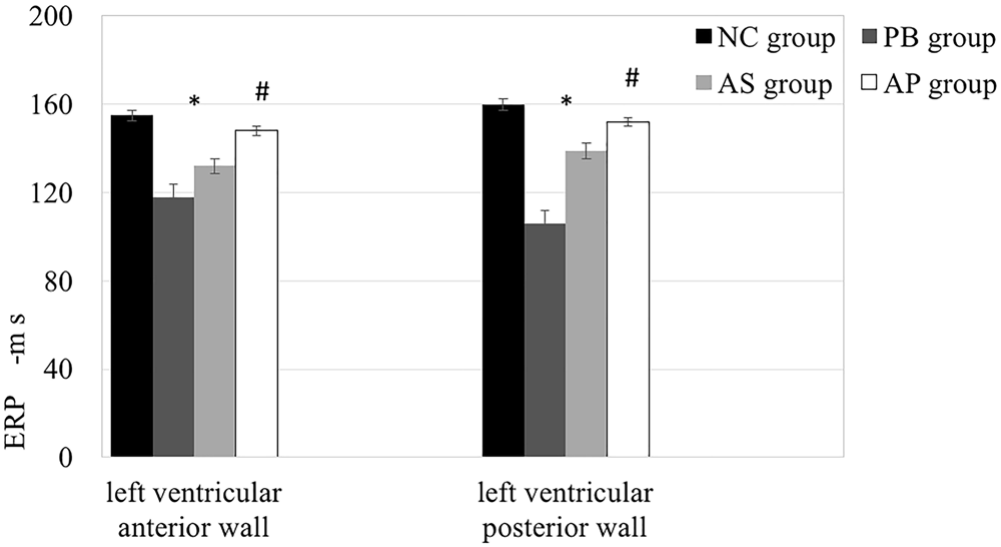
Ventricular effective refractory period (ERP) at the left ventricular anterior and posterior walls were measured after ES in PB, AS and AP groups. The animals in NC group didn’t undergo ES. ^*^p < 0.05, compared to NC group.

To determine potential effects in ventricular electrical activity conferred by ES, we monitored ERPs at the left ventricular anterior and posterior walls. Compared with the NC group, ES at the PB and AS sites significantly reduced the left ventricular ERP (anterior wall; PB group, 118 ± 4 ms; AS group, 132 ± 6 ms; AP group, 148 ± 4 ms; compared to NC group, 155 ± 4 ms; posterior wall; PB group, 108 ± 3 ms; AS group, 139 ± 6 ms; AP group, 152 ± 6 ms; compared to NC group, 160 ± 3 ms; ^*^p < 0.01; ^#^*p* > 0.05; Fig. 3). Although the ERP differences between PB and AS groups did not reach statistical significance, the changes of ERP in PB group have strong down trend. Next, we determined VFT in all animals at the end of electrophysiological study. The VFT in the PB and AS groups is much shorter than that in the NC group (PB group, 11.5 ± 1.5 V; AS group, 15.5 ± 0.5 V; compared to NC group, 20.5 ± 0.9 V; ^*^p < 0.01; Table 1). Summarily, ES at the different sites along the cardiac sympathetic nerves affects ventricular electrical activity are varied; moreover, the stimulation at the PB site induces the most drastic changes in ERP and VFT.

**Table 1:** ES and β1 -adrenergic receptor blockade regulate the ventricular fibrillation threshold (VFT).

	NC group	PB group	AS group	AP group
ES	20.5 ± 0.9	11.5 ± 1.5^*^	15.5 ± 0.5^*^	19.3 ± 0.8^#^
β1- blockade	25.8 ± 0.8	23.8 ± 2.1^#^	23.8 ± 0.8^#^	25.0 ± 1.2^#^

ES significantly shortened VFT in PB and AS group. The decreased VFT was reversed by β1-receptor blockade. *p < 0.05, #*p* > 0.05 compared to NC group.

**Figure 4:**
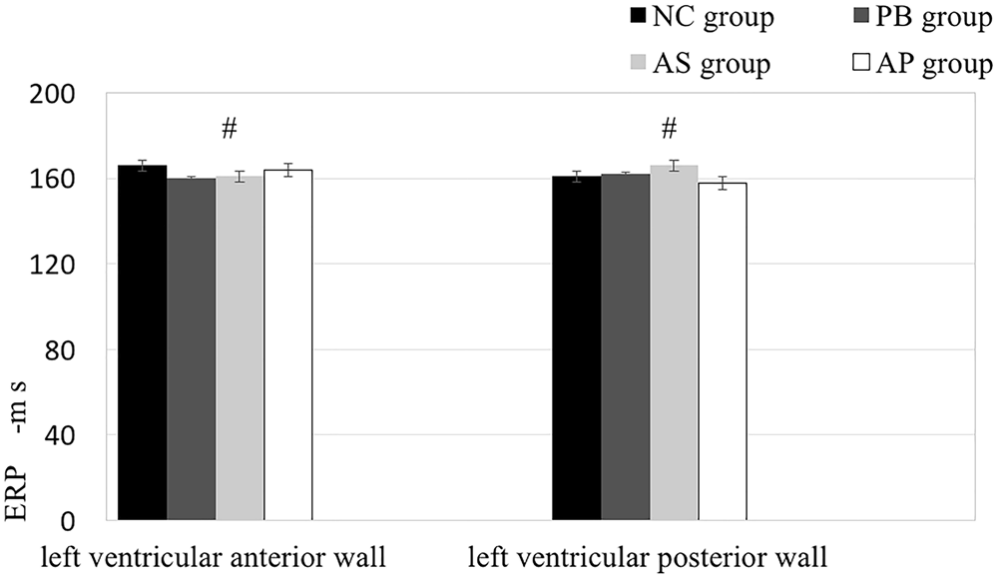
Ventricular effective refractory period (ERP) at the left ventricular anterior and posterior walls was measured after administration of β1-receptor blocker. ^#^*p* > 0.05, compared to NC group.

To explore the roles of the cardiac sympathetic nerves in ventricular electrical instability, we blocked beta-adrenergic receptors of sympathetic nerves with Esmolol prior to ES. As expected, β1-receptor blockade didn’t cause any changes in ERP and VFT in the animals undergoing ES (Fig. 4 and Table 1). This result indicates that the cardiac sympathetic nerves play a critical role in triggering ventricular electrical instability without obvious impact on heart rates among groups during the experiment.

### Examination of the regional cardiac sympathetic and parasympathetic nerves

**Figure 5:**
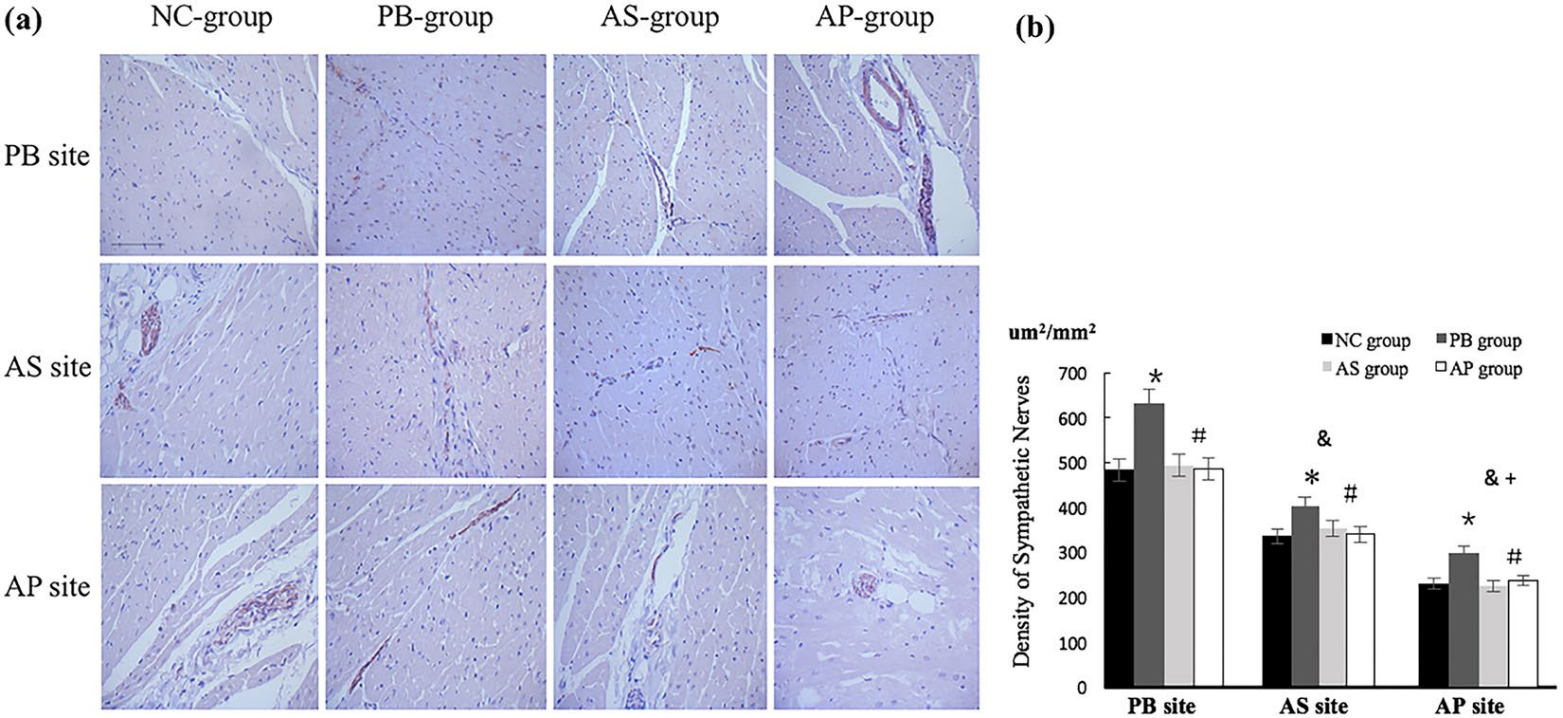
Density of sympathetic nerve fibers was demonstrated by IHC. ES at the PB site promoted the cardiac sympathetic nerves regeneration more obviously than other groups. Compared with untreated animals, ES at the AS and AP sites did not affect the density of TH positive nerve fibers in ventricular tissues. The density of sympathetic nerves numbers at PB site was highest among the observed sites. *p  < 0.05 compared with NC group. ^&^*p* < 0.05 compared with PB site. ^+^*p* < 0.05 compared with AS site. Scale bars, 200 μm.

**Figure 6:**
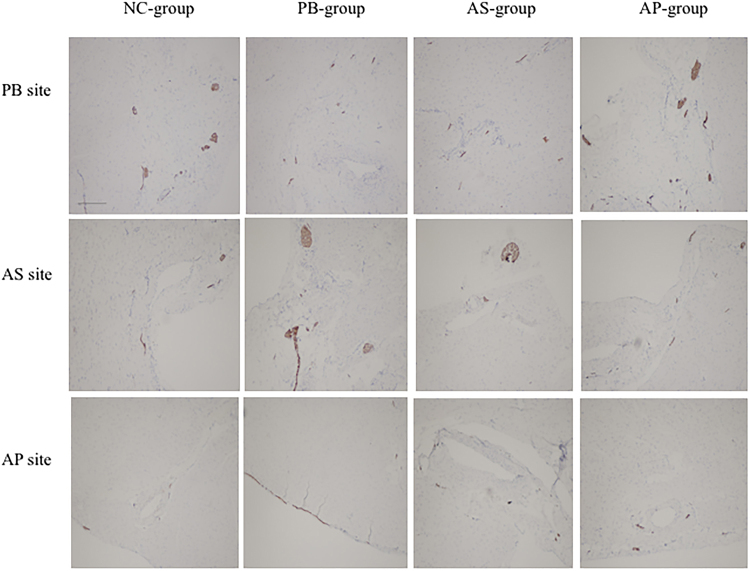
Density of parasympathetic nerve fibers was demonstrated by IHC. There was no difference in fiber density from base to apex. ES had no influence on the cardiac parasympathetic nerves regeneration among all experimental groups. Scale bars, 400 μm.

To determine whether the extent of electrical instability is correlated with the density of nerve fibers around ES sites and whether ES affects the regeneration of sympathetic nerves, we performed immunohistochemical for tyrosine hydroxylase (TH), a marker of sympathetic nerves (Fig. 5) and ChAT, the synthetic enzyme for the main parasympathetic neurotransmitter acetylcholine (Fig. 6). In the NC group, the TH positive nerves at the PB, AS and AP areas displayed distributional heterogeneity of highest to the lowest density, respectively. In the experimental groups, the cardiac sympathetic nerves were also distributed in the same pattern (Fig. 5). The PB area, at which ES triggered the most dramatic change of electrical instability, contained the highest density of nerve fibers, suggesting that the density of sympathetic nerves around ES sites is highly correlated to the degree of ES-induced electrical instability. Interestingly, compared with the groups of NC, AP and AS, the tissues from animals in the PB group contained more TH positive nerves at the PB, AP and AS sites. These results indicate that ES at the PB site, not at the AP and AS sites, stimulates regeneration of sympathetic nerves in the ventricle. A comparison of PB, AS and AP areas also showed that the ChAT-positive nerves in the ventricle were innervated heterogeneously, but they were different from the TH positive nerves. There was no difference in parasympathetic nerves density between PB, AP, and AS sites in the NC group, nor in the three experimental groups (Fig. 6).

## Discussion

In this study, we stimulated the cardiac sympathetic nerves at three different sites (PB, AP and AS) along its innervation and observed alterations in electrical activity of the left ventricle. ES at the different sites induced ventricular electrical instability of various grades, which were positively correlated with the density of sympathetic nerves around ES sites. Our study suggests that the anatomical heterogeneity of sympathetic nerves is highly related to cardiac electrical activity, and the epicardial area containing a high density of nerve fibers is a potential targeting site for modulating ventricular electrical activity.

It is well known that sympathetic nerves do not innervate evenly across cardiac tissues. The PB site serves as a major intermediate station for sympathetic nerve innervation from stellate ganglion to the heart, particularly to the ventricle^10–12^. The PB site is especially close to large diameter coronary veins (e.g., CS), and contains the highest density of sympathetic nerve fibers in the ventricle. ES at the BP site induces ventricular electrical instability to the maximum, as reflected by decreased ERP and VFT. These results demonstrate that electrical activity of the ventricle is tightly regulated by the heterogeneity of cardiac sympathetic nerves. In other words, modulating sympathetic neural tone at the PB site is an easier way to alter electrical activity of the left ventricle.

Increased sympathetic neural tone plays an important role in genesis and maintenance of ventricular arrhythmia (VA)^13–17^. Stellate ganglion stimulation shortens ventricular ERP and increases ventricular electrical activity instability^18–21^. However, how local stimulation of the cardiac sympathetic nerves influences electrical activity of the ventricle is not well studied. Our study found that ES at different sites along the cardiac sympathetic nerves induces electrical instability to various degrees. Furthermore, ES at the PB site causes the most significant electrical and anatomical remodeling in ventricular tissues, without impact on heart rate. In line with our observations, Meyer *et. al* showed that ES for sympathetic nerves inside CS does not change sinus node function and atrioventricular conduction, but selectively increases left ventricle (LV) contractility^6^. Although LV contractility was not measured in the present study, we speculate that an increase of LV contractility to varying extents would be found after ES at each of different locations, in a manner consistent with the degrees of change associated with electrical activity of the ventricle. The various degrees of electrical activity may also differentially affect the local concentrations of neurotransmitters, such as catecholamines and neuropeptides. The more active the sympathetic nerves, the larger amounts of neurotransmitters they secrete. This hypothesis is supported by our previous findings, in which local denervation of sympathetic fibers significantly reduced norepinephrine (NE) levels in CS blood^5^.

Sympathetic and parasympathetic nerves are in close proximity to each other in the atria and along the coronary sinus, and then traverse towards the ventricular apex via different routes, at which point the two types of nerves meet again and intertwine. Unlike sympathetic nerves whose distribution tightly follows the pattern of the coronary veins in cardiac tissues, the vagal nerve does not follow any specific pattern and is widely distributed in ventricular tissues^22^. Joseph *et al*. found parasympathetic innervation to be significantly concentrated in the ventricles^23^. However, in the present study, we observed the opposite, with a low density of the parasympathetic nerves in the ventricles, even within the PB site. Because the present study was conducted in canine hearts, whether this inconsistency is species related is not clear. It has been shown that epicardial parasympathetic denervation does not significantly affect the electrophysiologic properties of the ventricles^24,25^. Therefore, the ventricular sympathetic nerves play dominant roles of the ventricle especially since the sympathetic nerve fibers are bundled to increase density. This phenomenon can explain the difference in SBP responses when sympathetic nerves are stimulated at the different locations. Although SBP increased by more than 20 mmHg after ES, SBP in the PB and AS groups increased much faster than that did in AP group. Sympathetic nerves function dominates around PB and AS sites due to their high density, while the AS area has a relatively low density of sympathetic nerves, which results in a slower increase of SBP.

The high density of sympathetic nerves around the PB site enables efficient modulation of cardiac neural tone, thus influencing the occurrence of VA. In our previous studies^5,26^, we ablated the local cardiac sympathetic nerves at the PB site, proximal 2 cm from the CS ostium, in animals suffering AMI. Local denervation significantly prevents VA complicated with MI in those animals. Locally targeting sympathetic nerves is superior to other methodologies which modulate cardiac neural tone at locations far from heart, such as renal denervation and high thoracic epidural anesthesia. Our procedure does not generate the side effects inflicted by other methods. For example, blocking a stellate ganglion may cause Homer’s syndrome, difficulty in swallowing, vocal cord paralysis and pneumothorax, none of which we observed in our studies^27^.

In the present study, we showed that ES at the different sites along the cardiac sympathetic nerves triggers left ventricular electrical instability to various degrees. The stimulation at the PB site on the epicardium influences the electrical activity of the left ventricle to the greatest extent. Taken together with our previous study^5,26^, in which denervation at PB site can prevent VA complicating AMI, the PB site on the left ventricle may be the best site for locally intervening sympathetic nerves and for modulating the ventricular electrical activity.
